# Evolving Techniques and Trends in Maxillary Sinus Lift Procedures in Implant Dentistry: A Review of Contemporary Advances

**DOI:** 10.7759/cureus.71424

**Published:** 2024-10-14

**Authors:** Mohammed S Alsharekh, Afnan A Almutairi, Aishah S Jahlan, Alanoud S Alhazani, Sarah M Almohaimeed, Lamya A Aljnoubi, Ghadah A AlGhamdi, Taif T AlBenyan, Shatha F Alduhyaman, Najla M Alnaffaie, Abdulaziz M Altalhi

**Affiliations:** 1 Prosthodontics, Riyadh Elm University, Riyadh, SAU; 2 General Dentistry, Princess Nourah Bint Abdulrahman University, Riyadh, SAU; 3 Preventive Dental Science, College of Dentistry, King Saud bin Abdulaziz University for Health Sciences, King Abdullah International Medical Research Center, Ministry of National Guard Health Affairs, Riyadh, SAU; 4 Dentistry, Armed Forces Hospital, Riyadh, SAU; 5 Dentistry, Majmaah University, Riyadh, SAU; 6 General Dentistry, Batterjee Medical College, Jeddah, SAU; 7 General Dentistry, Dr. Abdulaziz Al Ajaji Dental Polyclinics Company, Dr. Sulaiman Al Habib Medical Group, Buraydah, SAU; 8 Dentistry, NEOM Community Advanced Health Center, Neom, SAU; 9 Dentistry, Ministry of Health, Riyadh, SAU

**Keywords:** dental implants, indirect sinus lift technique, maxillary sinus augmentation, schneiderian membrane, sinus lift surgery

## Abstract

The most challenging aspects of planning implant rehabilitation for the posterior maxillary region are the pneumatization of the maxillary sinus and the resorption of the residual alveolar ridge. To address the first aspect, there are two primary modalities for sinus augmentation: the direct or lateral window technique and the indirect or crestal technique. Clinicians must possess comprehensive knowledge of the maxillary sinus anatomy and be able to diagnose pathognomonic abnormalities through presurgical imaging and investigations. This skill is essential for performing technique-sensitive augmentation of the maxillary sinus with or without using biomaterials such as bone grafts, membranes, and platelet concentrates. These materials help create a suitable bone bed for either simultaneous or delayed implant placement through various traditional and evolving modified sinus augmentation techniques. It is also critical to effectively elevate the Schneiderian membrane during sinus floor elevation surgery, as complications in this process can adversely affect implant survival and potentially lead to implant failure. This review explores various aspects of maxillary sinus augmentation, ranging from traditional methods to evolving contemporary techniques, and draws on existing literature to examine their advantages, limitations, indications, and contraindications.

## Introduction and background

The maxillae are two bony structures in the facial skeleton that join at the mid-sagittal line via the intermaxillary suture [1]. Each maxilla houses a maxillary sinus, which is the largest of the paranasal sinuses [2,3]. The posterior maxilla is often devoid of bone volume due to two processes: centripetal resorption (the typical resorption of the residual alveolar ridge following tooth extraction) and pneumatization (the enlargement of air-filled sinuses, which leads to the thinning of the surrounding bone, particularly toward the crest of the ridge in the posterior maxilla, as seen in the case of the maxillary sinus) [4]. A sinus lift procedure involves the anterior, posterior, and medial displacement of the Schneiderian membrane. It aims to reduce the sinus boundaries and decrease its volume, thereby augmenting the residual alveolar bone volume in the posterior maxillary region. The primary objective is to establish a sufficient bony bed to support functionally stable dental implants, which may be achieved with or without incorporating grafting biomaterials [5,6]. The implant placement can be either a simultaneous one-stage placement during sinus elevation surgery or a delayed two-stage placement where the implants are placed at a later stage after sinus elevation surgery when the required bone quantity is formed [7].

In 1976, Tatum introduced the sinus lift surgical technique at the Alabama Dental Implant Conference [8]. Boyne and James later expanded on the technique and published their findings in 1980 [9]. Traditionally, there are two ways to execute sinus Schneiderian membrane elevation surgery: direct or lateral antrostomy surgery and an indirect or crestal technique. These strategies have developed over time and undergone various modifications and enhancements to improve their safety and effectiveness, particularly as minimally invasive techniques. They are often paired with a variety of biomaterials and blood-derived biologics.

This study aims to emphasize current, evidence-based practices and recommendations for sinus floor elevation and augmentation techniques, as well as their indications, contraindications, and procedural outcomes within the niche of implant dentistry in the posterior maxilla. Additionally, it delves into the use of innovative biological materials for sinus bone grafting, as supported by recent research.

## Review

Historical overview of sinus lift surgery

The practice of sinus floor elevation was established before the advent of contemporary dental implant technology. Maxillary sinus augmentation was already in progress during the 1960s, a few decades before Brånemark introduced biocompatible titanium-based implant root forms [10]. The sinus lifting performed in the past by Boyne was done to elevate the height of respiratory epithelium (i.e., the Schneiderian membrane) in view of shortening the height of the residual alveolar ridge or tuberosity. This would increase the inter-arch space between the crest of the alveolar ridge to accommodate traditional removable dentures [11]. The historical milestones of sinus augmentation surgery [9-21] are summarized in Table 1.

**Table 1:** Historical timeline of sinus augmentation surgery

Author and year	Milestones
Tatum (1976) [8]	Introduced the lateral sinus lift surgical technique at the Alabama Dental Implant Conference
Boyne and James (1980) [9]	Expanded the lateral sinus lift technique pioneered by Tatum and published their findings
Tatum (1970) [12]	Augmented the posterior maxillary region using an autogenous rib
Tatum (1974) [12]	Developed a modified Caldwell-Luc procedure by infracturing the crest of the alveolus
Summers (1994) [12]	Described another crestal approach sinus lift using tapered osteotomes with increasing diameters
Chen (1996) [12]	Provided the hydraulic sinus condensing technique
Tatum (1986) [13]	Described the transalveolar approach, also referred to as the transcrestal approach, as an alternative to the lateral window technique
Wood and Moore (1988) [14]	Reported the hinge osteotomy technique
Fugazatto (1999) [15]	Modified the trephine technique
Vercellotti et al. (2001) [16]	Described the piezoelectric bony window osteotomy and sinus membrane elevation
Soltan and Smiler (2005) [17]	Provided the antral membrane balloon elevation procedure
Pommer and Watzek (2009) [18]	Provided the gel pressure elevation sinus lift technique
Lozada et al. (2011) [19]	Described the Dentium Advanced Sinus Kit technique (Dentium)
Ahn et al. (2012) [20]	Described the reamer-mediated sinus floor elevation
Kher et al. (2014) [21]	Provided the minimally invasive transalveolar sinus approach

Types of sinus lift approaches

The Schneiderian membrane in the sinus cavity can be elevated toward the apex through various surgical approaches, such as transalveolar [8,22], lateral window [9], crestal window [23,24], or palatal window techniques [25,26], with the option to include bone grafts and implants during the same procedure if feasible. In modern dental implant dentistry, the lateral window technique, with or without immediate implant placement, is the most frequently utilized method for sinus lifts. Another commonly used approach is the transalveolar technique, which often allows for the simultaneous placement of implants [13]. The selection of a sinus lift technique by a surgeon for a specific patient is contingent upon the height of the remaining alveolar ridge and the extent of antral membrane elevation needed [27]. In cases where the posterior maxillary area is selected for rehabilitation with standard implant lengths (≥8 mm), sinus augmentation becomes essential when the remaining bone height (RBH) is compromised by a range of factors [28]. The requisite quantity of bone required for the positioning of standard-height dental implants is a minimum of 10 mm in vertical bone dimension and 4 mm in width of the alveolar ridge; sinus augmentation is needed for any measurement below this threshold [10,29]. The recommended dimension for a dental implant to get successfully osseointegrated to the bone is a minimum of 3 mm × 10 mm [2]. Therefore, during sinus lift surgery, the surgeon should primarily aim to have a vertical bone height of at least 12 mm after finishing the surgery, keeping in mind that the minimum length of the implant will be 10 mm. The few millimeters of excess bone created during augmentation will compensate for bone resorption during the healing period of approximately six months [30]. The criteria for direct versus indirect sinus lift indication are summarized in Table 2.

**Table 2:** Direct versus indirect sinus lift indication criteria

Sinus lift procedure	Bone dimension
Remaining bone height ≤3 mm	Maxillary sinus floor augmentation with a lateral window approach and delayed standard implant insertion
Remaining bone height of >3 to ≤5 mm	Maxillary sinus floor elevation utilizing a lateral window technique with immediate implant insertion or transalveolar maxillary sinus floor elevation with simultaneous placement of short implants
Remaining vertical bone height of >5 to ≤9 mm	Transalveolar maxillary sinus floor augmentation and simultaneous standard implant placement

Direct sinus lift technique

When the height of the residual alveolar ridge in the posterior maxillary region is reduced, typically with ≤5 mm of RBH and a need for at least 3 mm of vertical sinus bone augmentation, it is advised to utilize the lateral window technique [13,30]. The preparation of the bony lateral window can involve various devices, such as mechanical rotary bursts, advanced devices like piezoelectric bone-cutting handpieces, and hard tissue dental lasers [13]. In addition, there are scenarios where direct sinus lifting is predominantly preferred. For instance, sinuses with characteristic pathognomonic features, including polyps and multiple septa, as well as instances of reentry in a prior sinus lift surgery due to perforation [12].

Steps in the Lateral Window Sinus Lift Technique

An incision is made in the middle of the crest or toward palatally between the maxillary tuberosity and to a point anterior to the anterior boundary of the sinus. A vertical releasing incision is made on either side, extending into the vestibule past the mucogingival junction. A complete-thickness mucosal periosteal flap is elevated, exposing the lateral sinus wall. Cone beam CT (CBCT) shows the anteroposterior and superoinferior borders of the antral cavity. Four osteotomies (pairs of vertical and horizontal osteotomies) are performed linearly in brushing strokes on the lateral antral wall with a #6 or #8 diamond round bur starting from the inferior border within 2-3 mm above the sinus floor. The anteroposterior extent of osteotomy runs from the first or second molar posteriorly to the anterior border of the sinus. The osteotomy on the superior border is placed at the required height of the sinus bone augmentation. These two horizontal osteotomies are joined via two vertical osteotomies made parallel to the lateral nasal wall and maxillary tuberosity at the anterior and posterior extent, respectively. The safe stop for osteotomies is the appearance of a bluish or reddish hue on the borders of preparation, which indicates proximity to the Schneiderian membrane. The angles of the window are rounded. After window preparation, the quadrilateral-shaped bone over the exposed membrane is removed or rotated apicomedially into the sinus along with the membrane, creating a new sinus floor. Membrane elevation is initiated from the inferior, anterior, and posterior edges utilizing freers or curettes. It is important to raise the membrane just above the superior osteotomy to prevent tight packing of the graft beneath the membrane. Passively packing the graft promotes angiogenesis within the graft material [12,31,32].

Piezosurgery in the Lateral Window Sinus Lift Technique

The evolution of maxillary sinus lift procedures has introduced advanced techniques such as piezoelectric bony window osteotomy and piezoelectric sinus membrane elevation, which have demonstrated significant improvements over traditional methods. These techniques achieved a 95% success rate [16]. The piezoelectric bony window osteotomy procedure typically took about three minutes, while piezoelectric sinus membrane elevation required approximately five minutes, showcasing the efficiency of these methods compared to traditional rotary instruments, which have reported membrane perforation rates of 20-30% [16]. The precision of the piezoelectric bony window osteotomy is highlighted by the average dimensions of the bony window: 14 mm in length, 6 mm in height, and 1.4 mm in thickness. The approach utilizes piezoelectric devices that cleanly cut mineralized tissue while preserving the Schneiderian membrane’s integrity, significantly reducing the risk of complications. The advantages of piezosurgery include its precise and tactile properties, as well as the ability to cut only mineralized tissue, which mitigates soft tissue injuries [12].

Additionally, the study used autogenous bone grafts mixed with autogenous platelet-rich plasma gel. Postoperative assessments, including CT scans, confirmed the sinus membrane's integrity and graft stability. The combination of piezoelectric bony window osteotomy and piezoelectric sinus membrane elevation, along with the bone graft, not only simplifies the sinus augmentation process but also improves outcomes by reducing operative time, lowering complication rates, and enhancing bone maturation and vascularization [16]. These advancements make piezoelectric techniques a promising alternative to traditional sinus lift methods, offering improved safety, efficiency, and overall success in implant dentistry.

A study by Li et al. [33] explored the application of piezoelectric surgery combined with hydraulic pressure for maxillary sinus floor elevation, xenograft, and simultaneous implant placement in patients with insufficient residual alveolar bone. The average residual bone height was 3.7 ± 0.8 mm. This technique aimed to reduce surgical trauma and enhance predictability. The procedure utilized piezosurgery to elevate the sinus membrane without perforation, with hydraulic pressure further aiding membrane elevation. The technique employed intralift tips (TKW1-TKW5) to gradually widen the access canal to the Schneiderian membrane, ensuring minimal trauma. The mean sinus floor elevation achieved was 7.5 ± 0.9 mm. The study reported no implant failures over a follow-up period averaging 20.4 months, demonstrating significant gains in bone height from 3.7 mm preoperatively to approximately 11 mm postoperatively. The combination of piezoelectric surgery and hydraulic pressure effectively reduced trauma and complications, positioning this approach as a less invasive and reliable alternative to traditional sinus lift techniques.

Rickert et al. [34] conducted a randomized controlled clinical trial comparing piezoelectric devices with conventional rotary instruments in maxillary sinus floor elevation surgery. The study involved 36 patients who underwent bilateral sinus floor elevation using both methods in a split-mouth design. The results showed no significant differences in sinus membrane perforation rates between the techniques, with four perforations occurring in each group; however, the operation time was longer with piezosurgery (average 15.1 ± 2.9 minutes) compared to rotary instruments (average 11.1 ± 2.4 minutes) [34]. While piezosurgery offers selective cutting ability that reduces soft tissue injury, it does not significantly outperform conventional rotary instruments in preventing membrane perforations, and the increased operation time remains a notable drawback.

Antral Membrane Balloon Elevation (AMBE)

AMBE is a sinus lift technique that utilizes a minimally invasive approach to elevate the sinus membrane using a latex balloon attached to a metal shaft. The balloon is gradually inflated with saline solution, incrementally lifting the sinus membrane without the need for sharp instruments, which significantly reduces the risk of perforation and trauma compared to traditional methods. The technique is particularly advantageous in areas that are difficult to access, such as between adjacent teeth, and requires only limited incisions and minimal mucoperiosteal flap reflection [17]. The AMBE technique allows for controlled elevation to the medial wall of the sinus cavity, avoiding sharp dissection around adjacent tooth roots and minimizing complications like bleeding, infection, and postoperative pain [27,35]. Typically, 1 cubic centimeter of saline solution can achieve 5-6 mm of sinus floor elevation, making this method both effective and predictable [35]. The balloon is available in angled and straight designs tailored for lateral and crestal approaches, respectively [35]. Studies have demonstrated successful outcomes with this technique, showing minimal discomfort, reduced operative time, and a high success rate in simultaneous implant placements [35].

A novel method for sinus elevation utilizing a modified ballooning technique and a No. 6 Foley catheter through a hydraulic pressure mechanism was proposed by Sharanappa et al. [36]. This approach involves a lateral window technique for maxillary sinus augmentation, utilizing the Foley catheter to elevate the sinus membrane by hydraulic pressure. The study demonstrated an increase in alveolar bone height of 7.8 mm from a preoperative residual ridge height of 3.0 mm, achieving sinus floor elevation of up to 7 mm. The technique was particularly noted for its cost efficiency and minimal invasiveness, as it uses easily accessible tools and reduces the need for traditional osteotomes and mallets, which are often associated with adverse effects such as auditory and equilibrium disturbances. Additionally, the method eliminates the need for special instrumentation, making it accessible in resource-limited settings. Despite its advantages, the procedure requires careful handling to avoid membrane tears, and the inability to monitor internal balloon pressure poses a risk of overinflation [36]. A modified version of AMBE, minimally invasive AMBE (MIAMBE), involves a transcrestal approach using an inflatable balloon device through a 3-mm osteotomy site, which will be discussed in the forthcoming sections [37].

Hydraulic Pressure for Maxillary Sinus Augmentation

The use of hydraulic pressure for maxillary sinus augmentation has been evaluated as an effective method for sinus membrane elevation through a lateral approach with simultaneous implant placement [38,39]. The procedure utilized a lateral window technique with a specialized water lift system, which applied equal pressure on the Schneiderian membrane to gently elevate it without the risk of tears. Saline was injected through the osteotomy site to achieve membrane elevation, followed by grafting. Clinical outcomes showed a significant increase in subantral bone height from 3.86 mm to 15.49 mm after six months, with no membrane perforations or significant complications observed, demonstrating the predictability and safety of the technique [38,39].

The evolving landscape of maxillary sinus lift techniques reflects a shift toward minimally invasive and highly controlled approaches, such as piezosurgery and balloon-assisted sinus elevations. These methods not only minimize the risk of complications, such as membrane perforation, but also enhance patient comfort and reduce recovery times. The clinical outcomes reported across various studies consistently demonstrate the effectiveness of these advanced techniques in achieving predictable bone height gains and high implant survival rates. However, the variability in procedural time, equipment accessibility, and the technical skill required may influence the adoption and success of these techniques in clinical practice. The integration of technologies like hydraulic pressure systems and guided surgical approaches further emphasizes the importance of precision and safety in sinus augmentation, suggesting that continued innovation and refinement of these methods could set new standards for implant dentistry.

Guided Sinus Lift Techniques

The integration of guided sinus lift techniques using 3D printed templates and digital planning has significantly enhanced the precision and safety of sinus augmentation in complex cases. One proof-of-concept case report demonstrated the use of 3D printed surgical guides for the lateral sinus lift procedure, allowing for precise window creation and implant positioning that minimized the risk of complications, such as sinus membrane perforation and misaligned implant placements [39]. This method employs a fully digital workflow, utilizing CBCT scans and treatment planning software to create patient-specific guides that ensure the accurate execution of the surgery, ultimately improving functional and aesthetic outcomes. Additionally, a retrospective clinical study on guided implant surgery combined with lateral sinus lift augmentation in severely resorbed maxillae underscored the long-term success of this approach. Over a follow-up period averaging 5.11 years, the study reported no implant failures, minimal complications, and significant bone height gains from 2.07 mm to 12.83 mm. The digital approach allowed precise graft placement and implant positioning, reducing the potential for errors during surgery and enhancing overall implant stability and success rates [40]. This evidence highlights the growing role of guided techniques in advancing sinus lift procedures, offering a more predictable and less invasive alternative for managing complex anatomical challenges in implant dentistry.

Indirect sinus lift technique

This approach of sinus lift is less invasive and more conservative, as it avoids cutting a large bony window. This technique is also called the Summers osteotome technique, crestal technique [27], transalveolar or transcrestal technique [13], and sinus intrusion osteotomy technique [31]. It is recommended for residual vertical bone heights of >5 mm, and bone heights of 3-4 mm can be gained [41]. In 1994, Summers [22] introduced a modified version of Tatum’s original 1986 transalveolar technique by using tapering osteotomes of increasing diameter [22]. This method has passed through various modifications over the decades, and in recent years, it is a form of advanced and minimally invasive approach that is better accepted among patients.

Steps in the Indirect Sinus Lift Technique

A crestal incision is performed following the administration of anesthesia, and a full-thickness mucoperiosteal flap is elevated to reveal the crest of the alveolar ridge. In cases where an autogenous graft needs to be obtained, this crestal incision is extended distally to the tuberosity. A 2 mm-diameter pilot drill is employed for the preliminary drilling, stopping 2 mm before reaching the sinus membrane. This is verified with a confirmatory periapical radiograph while the pilot drill remains in position.

If the residual alveolar bone is soft or the vertical residual alveolar bone height is 5 mm or less, pilot drilling is not performed. The complete procedure is accomplished with a series of sequential osteotomies of increasing dimension, and the osteotomy site is widened by controlled malleting of the osteotomes 2 mm short of the sinus floor. In cases where the nature of the bone is soft (D3 or D4), controlled malleting of the concave tip of the osteotome exerts an apical and lateral pressure that causes the bone to move upward toward the sinus floor while compacting laterally improves the bone density.

The trapped bone debris and fluid during the osteotomy, combined with the constant, controlled pressure from malleting the osteotomy, push and flex the sinus floor upward, thereby slowly elevating it. Before tapping with the final osteotome, which is 0.7-1 mm in diameter short of the planned implant diameter, the desired bone graft material is added in a hydrated slurry form to the osteotomy site. The final osteotome gently fractures the sinus floor, providing a different pitch sound during malleting. The bone graft, which is augmented in the osteotomy site, exerts pressure and elevates the sinus further. The implant diameter is slightly ahead of the final osteotome diameter to provide better implant primary stability [3,42,43].

Modifications and Evolving Minimally Invasive Techniques in the Crestal Sinus Lift Approach

In his initial sinus lift procedures, Summers [22] conducted elevation of the sinus floors using osteotomes through a malleting technique known as osteotome sinus floor elevation (OSFE). Subsequently, he integrated the hydrated bone graft slurry into this method, referred to as bone-added OSFE. Summers further used a larger-dimension osteotome to penetrate the ridge crest and create a wider area for sinus elevation. Additionally, he emphasized the importance of meticulous packing of bone grafts in the osteotomy site to establish a robust volume of bone, which would facilitate the subsequent placement of implants. This process is referred to as future site development [12,22].

Fugazzotto executed the modified trephine/osteotome approach, in which a 3 mm outer diameter trephine is inserted until it is 1-2 mm short of the sinus floor. Following this, an osteotome is employed to apically displace the bone cylinder, accompanied by concurrent implant insertion [15].

MIAMBE follows the same initial steps as the crestal approach sinus lift, like flap elevation, pilot drill osteotomy, and a series of sequential osteotomes drilled to a depth 1-2 mm short of the sinus membrane. Next, the sinus floor is gently fractured and the integrity of the sinus membrane is checked. A dedicated inflatable balloon is inserted into the osteotomy site and inflated at 2 atm pressure via a dedicated inflating syringe using a diluted contrast solution, which is useful in radiographic detection. Once the desired membrane elevation is attained, the balloon is deflated and removed. Later, the bone grafts are packed beneath the sinus floor, and appropriately sized implants are screwed into the osteotomy site [37-99]. A recent randomized clinical trial by Alajami et al. [99] compared the antral membrane balloon technique to the Densah bur technique for crestal sinus lift procedures with simultaneous implant placement. The balloon group demonstrated a higher immediate postoperative vertical bone gain, achieving an average of 8.31 mm compared to 6.75 mm in the Densah group. Despite the initial differences, the bone height gain between both groups became statistically insignificant after six months. Importantly, no membrane perforations were reported in the balloon group, underscoring the safety and predictability of this minimally invasive approach [99].

The hydraulic pressure technique was adopted in 2005 by Sotirakis and Gonshor [44] as a modification of the summer technique. After the use of osteotomes, saline is infused inferior to the membrane under hydraulic pressure to elevate the sinus membrane. Another method using a similar high-pressure (1.5 bar) technique is known as minimally invasive sinus floor augmentation and was developed by Jesch et al. [45].

A method of the crestal approach developed by Chen [46], known as the hydraulic sinus condensation method, involves using a 3 mm sinus diamond bur positioned 1 mm below the sinus floor, followed by a 2 mm downsized sinus bur to create a conical shape at the apex of the osteotomy. Hydraulic pressure is introduced through this conical entry from the high-speed handpiece, which separates the sinus membrane from the floor without perforation, allowing for graft packing with an appropriately sized condenser [46,47]. This method carries the risk of air embolism but offers the advantage of atraumatic sinus membrane elevation without perforation.

In 2009, Pommer and Watzek [18] conducted the gel pressure sinus elevation technique. This method utilizes a mixture comprising a viscoelastic agent (2% hydroxypropyl methylcellulose) and a radio-opaque agent (37% iopamidol) in a 3:1 ratio, delivered in gel form through a specially designed injection nozzle for sinus elevation [18]. A specialized reamer with an 85-degree cutting-edge angle (CEA) accompanied by a flat end on the other side was used by Ahn et al. [20] for sinus elevation. In this reamer-assisted sinus elevation, the 85-degree CEA prepares the osteotomy site. Then, the reamer is operated at a slow speed of 30-50 rpm with bone grafts to elevate the sinus membrane, while the flat end exerts an upward pushing force on the sinus membrane, detaching it from the underlying floor [20].

Cosci and Luccioli [48] described a technique for performing a crestal sinus lift as a single-stage intervention that employs atraumatic drilling instruments of diverse dimensions. This methodology effectively mitigates the risks of sinus floor fracture and perforation. The procedure involves systematically drilling the cortical bone situated inferior to the sinus floor in a deliberate and controlled abrasive manner [48]. Upon achieving the requisite height, a blunt-tipped instrument is utilized to assess the osteotomy site through tactile feedback of the sinus membrane.

Kher et al. [21] performed a transalveolar sinus lift approach using a calcium phosphosilicate putty consistency graft (novabone putty) and called it a minimally invasive transalveolar sinus approach. After sequentially inserting the drill bits, the osteotomy site is prepared 1 mm short of the sinus floor, and a 3 mm concave tip osteotome is utilized to make a green stick fracture on the floor of the sinus. The NovaBone gun cannula is then inserted into the osteotomy site with a snug fit. The graft material is deposited, which lifts the sinus membrane by hydraulic pressure mechanism due to its consistency [21]. In another study, Pozzi and Moy proposed computer-aided design and manufacturing of surgical guides for planning implant placements. They recommended a transcrestal approach along with the use of expander-condensing osteotomes in a minimally invasive manner [49].

Osseodensification is a bone non-extractive or non-subtractive drilling technique introduced by Huwais in 2013 [50]. This method creates plastic deformation of bone through rolling and sliding contact using densifying burs (DENSAH burs). The flutes of DENSAH burs are designed to densify and compact the bone while minimizing heat generation as they prepare the implant site [50]. Traditional implant osteotomy drills remove bone during preparation, but DENSAH burs preserve bone by laterally condensing and compacting it, thereby enhancing bone density and bone-to-implant contact (BIC) [47,50]. Increased bone density, in turn, raises insertion torque, improving primary stability and eliminating micromotion. This technique was initially employed for the horizontal augmentation of thin ridges, but it also facilitates the acquisition of vertical height through a transcrestal approach [47,51].

During this process, the DENSAH drill bits operate at 800-1500 RPM in an anticlockwise direction, utilizing a dedicated external irrigator. A slurry of hydrated bone graft is added to the osteotomy site if further sinus elevation of 3 mm or more is required. The final diameter DENSAH bur runs at 150-200 RPM without irrigation in a counterclockwise direction, compacting the graft apically and elevating the sinus. The final drill diameter is typically 0.7 to 1 mm shorter than the planned implant diameter [51,52].

In a study conducted by Alajami et al. [99], the Densah bur technique was shown to offer superior primary implant stability compared to the balloon method, with statistically higher insertion torque immediately postoperatively. The Densah technique also led to significantly increased buccal and palatal bone density at both the immediate postoperative stage and after six months. These findings reinforce the advantages of osseodensification [50] in enhancing bone quality and implant stability, making it a valuable option for achieving long-term success in sinus lift procedures [99].

The Dentium-Advanced Sinus Kit System (DASK) via the transcrestal approach utilizes dome-shaped drills. This technique follows the same steps as other approaches, employing a series of sequential drill bits to create an osteotomy site with the desired diameter and 1 mm of bone left inferior to the antral floor. It is then followed by the unique dome-shaped DASK drill, which safely abrades the thin sinus floor bone beneath the Schneiderian membrane to avoid perforation. The sinus membrane is elevated with antral curettes, and bone grafts are packed below the sinus membrane [19,43,47].

The vertical expander screw (VES) technique was proposed by Kadkhodazadeh et al. [53] and involves employing the threaded expander in the osteotomy site via the transcrestal approach. The initial osteotomy is done 1 mm inferior to the sinus floor. Later, the osteotomy is widened and the sinus floor is gradually elevated apically with sequential increases in the size of the threaded expander until the requisite dimension is attained for final implant placement [53].

These evolving techniques in the crestal sinus lift approach represent significant advancements in minimizing complications and improving clinical outcomes. A key focus across methods has been the reduction of membrane perforation, with innovations like MIAMBE, hydraulic pressure techniques, and osseodensification offering improved control during sinus membrane elevation. These methods have successfully reduced perforation rates to as low as 5%, particularly with hydraulic pressure methods. Additionally, osseodensification has introduced a paradigm shift by preserving bone bulk during osteotomy preparation, which enhances both primary and secondary implant stability. The increase in BIC and insertion torque leads to faster recovery and higher long-term success rates. Furthermore, minimally invasive approaches such as the Dentium-Advanced Sinus Kit (DASK) and VES techniques ensure that even patients with limited residual bone height can undergo sinus elevation with reduced surgical trauma and quicker healing times. Collectively, these innovations have not only improved the predictability and safety of crestal sinus lifts but also provided clinicians with flexible, patient-friendly options for addressing complex clinical cases.

Bone grafts and biomaterials in sinus augmentation

Based on their origin, bone grafts can be categorized into four variants: autograft, allograft, xenograft, and alloplast. When the donor and recipient are the same, the graft obtained is called an autograft. A graft obtained from the same species but from a different individual is referred to as an allograft, while a graft from a different species is known as a xenograft. Alloplasts are grafts that are engineered from synthetic and natural materials [47]. Alloplastic substances include beta-tricalcium phosphate, bioactive glass, and calcium sulfate [53]. The purpose of grafting is linked to three biological mechanisms: osteogenesis, osteoinduction, and osteoconduction. Osteogenesis is the capability of the graft to bring about vital neo-bone formative cells to the recipient site. Osteoconduction is the ability of the graft matrix to function as a scaffold for bone cells to occlude and divide. Essentially, it allows the ingrowth of new blood capillaries, while osteoblasts from the periphery of the defect site utilize the scaffold framework for cellular migration and enhance the process of neo-bone development [54]. Osteoconductivity is an indispensable characteristic of a graft, as it offers a stable biological environment for blood clots in the early phase of healing and as a scaffold for new bone that forms in the later phase of healing [55]. Osteoinduction refers to the inherent capacity of the molecules present in the graft to stimulate resident undifferentiated osteoprogenitor cells to differentiate into osteoblasts, resulting in new bone formation [54].

Choosing the right graft for sinus augmentation surgeries is a crucial aspect of implant pre-planning. The earliest documented graft material for maxillary sinus floor grafting was autogenous bone [9]. Autografts can be sourced in two ways, either intraoral or extraoral. Intraoral sources include the tuberosity, ramus, and symphysis, and extraoral sources include the iliac crest, cranium, and tibia [56]. The anterior iliac crest (50 cc of corticocancellous bone) extraorally [2], and mandibular symphysis intraorally [57], provide large quantities of bone. However, there are a few disadvantages to obtaining this graft. Firstly, the amount of bone graft required varies from 0.5 cc to 5 cc [58], and it has to be sourced from a second distant surgical site. In addition, particulate autogenous bone is associated with a fast resorption rate [59], which exceeds the rate of new bone formation during the consolidation phase, resulting in reduced bone formation. Some authors [60,61], suggested using a combination of grafts, such as 20% autografts with 80% bovine xenografts or cortical allografts. This approach will integrate the benefits of autografts, such as osteogenicity, osteoinductivity, and osteoconductivity, with the low resorption rate of alternate bone grafts (xenografts and allografts) and thereby mitigate the disadvantages of autografts mentioned earlier [58,59]. Traditionally, in indirect sinus lifts, composite bone grafts consisting of 25% autogenous material and 75% hydroxyapatite grafts were used in practice [42].

Allografts can be either mineralized or demineralized and are obtained from cadavers in tissue banks. The mineralized form of allografts is often avoided in sinus augmentation surgery due to the extended time required for bone formation. In contrast, the demineralization process of allogenic bone reveals bone morphogenetic proteins (BMP) on the surface, which promotes osteoinduction in nearby undifferentiated cells and therefore enhances bone formation at the recipient site. The high cost associated with the demineralization process and the minimal risk of disease transmission are drawbacks of this type of allograft [56].

The BMP was first identified in 1960 by Urist [62]. Among the 15 BMPs, BMP-2 and BMP-7 have been extensively studied. The advancement of aminoacid sequencing techniques and recombinant DNA technology has facilitated the extraction and detailed investigation of these proteins. By utilizing these methods, researchers can determine the precise aminoacid sequences of BMPs and produce them in a controlled environment [62].

Currently, recombinant human BMP (rhBMP)-2 and rhBMP-7 are utilized instead of bone grafts, as they avoid the morbidity associated with a second surgical site, enhance soft tissue healing, and offer superior handling characteristics, making them advantageous for patients in whom autogenous grafts cannot be harvested. BMP is available as a lyophilized powder that is then mixed with sterile water and loaded into a resorbable carrier, likely collagen, in the case of sinus augmentation. The purpose of this carrier is to serve as a scaffold that retains rhBMP at the augmentation site and promotes osteogenesis [31,63].

Commercially available xenografts (Bio-Oss) undergo chemical processing that eliminates organic matter, resulting in deproteinized and inorganic cancellous bone components, which prevents immunogenic graft rejection. It regenerates a spongy bone architecture akin to human bone and follows a slow physiological bone remodeling process. As Bio-Oss possesses osteoconductive properties, it is frequently utilized in sinus lifting [53,64].

Former studies have noted that there is evidence of new bone formation and osseointegration of implants following sinus lift procedures without the use of grafting [65-67]. The expression of BMP and growth factors from the blood clot at the osteotomy site, which occurs due to bleeding from tissue injury caused by implant drills, along with the osteoprogenitor cells in the Schneiderian membrane, promotes bone formation [68]. The interaction between the blood clot and the titanium surface of the implant leads to thrombin activation and subsequent osteoblast activation, resulting in increased bone growth [69]. Lundgren et al. noted involuntary vertical osseous bone growth following the surgical removal of a cyst in the maxillary sinus region and emphasized the intrinsic osteogenic properties of the clot formed within the sinus. It has been reported that sinus elevation can increase bone height by 3.8 mm without the need for grafting [71].

Healing after sinus grafting typically takes about six to eight months, which includes the period from graft placement to bone neoformation and maturation [72]. Several factors influence healing following sinus grafting, such as residual vertical bone height, type of graft material, width of the sinus cavity, patient age, smoking habits, and iatrogenic sinus perforation [6]. The greater the sinus floor height from the bone crest, the shorter the healing period, the lower the incidence of complications, and the higher the success rate of sinus grafting and implant osseointegration [73]. In addition, a narrower sinus cavity width correlates with a better prognosis for sinus grafting [74].

The use of biologics is backed by limited positive evidence, as outlined in the 2022 Consensus Report from the American Academy of Periodontology [75]. Biologics comprise autologous blood-derived products (such as platelet-rich fibrin), enamel matrix derivatives, recombinant human platelet-derived growth factor-BB (rh-PDGFBB), and rhBMP2 [75]. They can be used either as a standalone therapeutic approach or alongside bone graft materials for implant site preparation, including sinus augmentation procedures. Biologics enhance bone formation and maturation while facilitating the healing of soft tissue, particularly in patients with co-morbidities such as diabetes mellitus and osteoporosis, where healing is often impaired [76]. Injectable platelet-rich fibrin (I-PRF) is a modified version of PRF. When combined with particulate bone graft, this autologous blood-derived biologic creates a polymerized graft mass known as “sticky bone,” which has enhanced manipulative characteristics and promotes local hemostasis [13]. This PRF improves implant stability in maxillary sinus floor augmentation [77]. Further long-term studies are needed to evaluate the efficacy of biologics and bone grafts in sinus augmentation procedures. Mononuclear stem cells (MSC) regenerate bone at a rate that matches the clinical effectiveness of autografts [78]. One randomized controlled trial implanted a BioOss bone graft with MSCs sourced from the posterior iliac crest to enhance bone formation following sinus augmentation surgery [79].

In a systematic review by Ghodsian et al. [81], autologous tooth grafts were found to be a safe and accessible alternative for sinus augmentation. They demonstrate superior clinical outcomes compared to autogenous bone and other graft materials. Furthermore, they are more cost-effective and biocompatible, exhibiting fewer complications than alternative biomaterials. Nonetheless, the review emphasized the necessity for additional long-term clinical studies with homogeneous data to elucidate the specific characteristics of autologous tooth grafts [80]. Paetnukroh et al. [82] conducted a randomized clinical trial comparing deproteinized human demineralized tooth matrix (dpDTM) and deproteinized bovine bone mineral (DBBM) for sinus floor augmentation with simultaneous implant placements. They found comparable volumetric graft changes (dpDTM -120.33 ± 77.48 mm^3^ and DBBM -108.51 ± 65.15 mm^3^) and implant stability between the two groups (implant stability quotient score 70 at six months). The study indicates that dpDTM could be a viable alternative to DBBM for this procedure [81]. In their retrospective analysis, Choi and Sohn [83] found that demineralized block-type autogenous tooth bone graft material resulted in a proportional improvement of alveolar bone height relative to the size of the bone graft, along with long-term stability during maxillary sinus augmentation procedures. The graft exhibited exceptional bone induction and biocompatibility while eliminating the drawbacks associated with autogenous grafts [83].

Discussion

Implants placed using indirect osteotome sinus elevation and direct sinus floor elevation methods demonstrate a 100% success rate, as shown by clinical and radiographic evaluations of immediate and delayed implant placements [73]. There was a slight difference in implant survival rates depending on the RBH; RBH >4 mm had a slightly higher survival rate than RBH ≤4 mm [84]. Furthermore, immediate implant placement depends on achieving primary stability, with no statistically significant difference in primary stability observed between the two methods. Implants may be delayed for up to six months if primary stability is not attained, and at least 3-4 mm of remaining bone is required for primary stability in a single-stage lateral approach sinus lift [71].

In a comparative study on direct and indirect sinus elevation techniques, Zitzmann and Scharer et al. recorded a similar range of sinus elevation height postoperatively in both immediate (10 mm) and delayed implant placement (12.7 mm) following direct sinus lift [85]. Another comparative study by Pal et al. noted that the indirect osteotome approach provided a bone height of 4.4 mm between the bone crest and the sinus floor, while the direct sinus lift approach provided a significantly greater sinus elevation height of 8.5 mm [86]. Balaji, in his comparative study, reported a higher gain in bone height from the direct sinus lift technique (6.19 mm for direct vs. 5.34 mm for indirect) [87]. Generally, the direct approach provides a greater sinus elevation than the indirect method. According to Khandelwal et al., the mean gain in bone height at three months following direct sinus augmentation and simultaneous implant placement was 8.31 mm [88]. Additionally, Al-Dajani, in his review, cited that 9 mm of vertical bone height can be achieved post-lateral antrostomy procedure [5]. However, adverse effects like post-surgical pain [89] and swelling [90] are more pronounced in the direct approach compared to the indirect approach, which gradually wears off one week after surgery.

The most common complication encountered during sinus elevation surgery is perforation of the Schneiderian membrane [13,71]. Membrane perforation can occur in any type of sinus lift approach, but the lateral approach often results in sinus membrane perforation in one out of five cases [91,92]. Minimal perforations within 2 mm are allowed to heal on their own [93,94]. A longitudinal study involving 359 sinus lift procedures conducted by Nolan et al. reported that after three years of observation, sinus membrane perforation was noted in seven out of every 10 cases of failed sinus augmentation [95]. In lateral window sinus lift procedures, 24% of Schneiderian membrane perforations are associated with the use of rotary burs, while piezosurgery accounts for 8% of membrane perforations [95]. Accordingly, researchers have developed minimally invasive techniques to address the issue of membrane perforation.

In a multicentric study by Kfir et al., the MIAMBE technique was performed on 109 subjects, and three of them encountered membrane perforation with a 95% implant success rate [97]. According to Chen and Cha [46], sinuses with complex anatomical configurations, such as sloping sinuses and compartmentalized sinus septa, can be effectively treated using hydraulic sinus condensation techniques. However, the use of a fluid jet increases the risk of perforation as it may cause pressure surges [47]. Pommer and Watzek [18] conducted a study utilizing the gel pressure sinus lift technique on ten human cadavers and concluded that the gel serves a cushioning function. By absorbing abrupt pressure and redistributing force across a larger surface area, it mitigates the risk of membrane perforation [18].

Endoscopic intrasinusal intervention revealed that when the transcrestal approach is performed meticulously within advantageous sinus anatomical configurations, as much as 5 mm elevation in the sinus floor can be achieved without compromising the integrity of the membrane. Endoscopy was also used for dislodged implant retrieval via the transnasal approach.

A clinical study compared Densah burs with AMBE via the crestal approach and simultaneous implant placement. It was found that the Densah group demonstrated improved bone density and implant stability, while the crestal AMBE approach exhibited immediate postoperative vertical bone height [97]. In a systematic review and meta-analysis comparing osseodensification and the osteotome technique for intracrestal sinus lift, the osseodensification method showed higher primary implant stability, with no significant difference in the mean gain in bone height [98].

Densah burs are regarded as a safe and effective method for performing crestal approach sinus elevations with simultaneous implant placements in oblique sinus floors that have 4-7 mm of residual bone height [99]. Although osseodensification burs are safe in terms of preserving membrane integrity, a multicentric study involving 621 patients revealed that the crestal approach sinus lift with osseodensification burs resulted in membrane perforation in 7.3% of cases (49 subjects). The majority of perforated cases had a residual bone height of ≤3 mm, followed by >3 mm and ≤5 mm [100].

In a comparative study of osseodensification versus lateral antrostomy sinus lift techniques in cases with RBH ≤ 4 mm, both showed similar effectiveness in simultaneous implant placement. However, the osseodensification in the crestal sinus lift approach demonstrated better outcomes in terms of pain perception, quality of life, surgery time, postoperative edema, and painkiller consumption. Further long-term studies with larger populations are needed to explore the advantages of osseodensification in the crestal sinus lift approach [101].

## Conclusions

From the above review, it is evident that there are two widely practiced approaches to sinus floor elevation, namely the direct and indirect techniques, with the lateral sinus lift technique being the most commonly employed. The lateral approach offers greater vertical bone height (6-12 mm) after sinus elevation surgery compared to the crestal approach. However, the direct sinus lift often poses a risk of Schneiderian membrane perforation due to its technique-sensitive nature and the surgeon's need for experience in meticulously lifting the sinus membrane via the lateral approach while understanding complex anatomical structures. To address this complication, numerous modifications of the intracrestal sinus lift in a minimally invasive manner have evolved. The intracrestal sinus lift can elevate the sinus by up to 5 mm. Sinus elevation without grafting can increase bone height by up to 3.8 mm. In cases with residual bone height of less than 3 mm, where the lateral approach is preferred, it is advisable to prepare the lateral bony window using a piezosurgery system due to its precision and hallmark characteristic of cutting only the bone, thereby minimizing the risk of membrane perforation.

Currently, autogenous bone grafts remain the gold standard for sinus augmentation and offer reliable osteogenic potential. However, some studies have suggested the use of combination grafts to address the limitations of autogenous grafts. The incorporation of biologics has proven to be an excellent adjunct to these grafts, as they enhance the grafts’ manipulative characteristics while improving soft tissue healing and bone neo-formation. Autogenous tooth grafts and the induction of stem cell regeneration in sinus augmentation can serve as viable alternatives to autogenous bone grafts, addressing their limitations. Further long-term homogeneous clinical trials are necessary to thoroughly explore the advantages of bone grafts and biologics in sinus augmentation surgeries. Healing after sinus grafting typically takes about six to eight months. The decision between one-stage or two-stage sinus augmentation with implant placement depends on primary stability. The osseodensification method of crestal sinus lift provides greater primary stability and bone density. The choice of either direct or indirect sinus augmentation is determined by the RBH. Given the various minimally invasive modalities of the transcrestal approach and the emergence of CAD-CAM-aided surgical templates for sinus lift procedures, the selection criteria for the method of surgical approach depends on the patient’s status, sinus anatomy, clinician’s experience, time, and the cost involved in the procedure. Accordingly, the use of piezosurgery, bone grafts, and biologics primarily relies on the clinicians’ decision-making skills and clinical abilities.
